# Microarray Analysis: Genome-scale hypothesis scanning

**DOI:** 10.1371/journal.pbio.0000015

**Published:** 2003-10-13

**Authors:** Greg Gibson

## Abstract

Microarrays can survey genome-wide expression patterns. Not only can these gene expression profiles be used to identify a few genes of interest, they are now being creatively applied for hypothesis generation and testing

Microarrays are used to survey the expression of thousands of genes in a single experiment. Applied creatively, they can be used to test as well as generate new hypotheses. As the technology becomes more accessible, microarray analysis is finding applications in diverse areas of biology. Microarrays are simply a method for visualizing which genes are likely to be used in a particular tissue at a particular time under a particular set of conditions. The output of a microarray experiment is called a “gene expression profile.”

Gene expression profiling has moved well beyond the simple goal of identifying a few genes of interest. The notion that this is the major objective of microarray studies has engendered the oft-repeated criticism that the approach only amounts to “fishing expeditions.” The sophistication of microarray analysis very much blurs the distinction between hypothesis testing and data gathering. Hypothesis generation is just as important as testing, and very often expression profiling provides the necessary shift in perspective that will fuel a new round of progress. In many gene expression profiling experiments, the hypotheses being addressed are genome-wide integrative ones rather than single-gene reductionist queries. In general, without a hypothesis only the most obvious features of a complex dataset will be seen, while clear formulation of the scientific question undoubtedly fuels better experimental design. And in some cases, the results of a microarray screen that was initially designed as an effort at cataloguing expression differences are so unexpected that they immediately suggest novel conclusions and areas of enquiry.

## Fundamental Microarray Technology

All microarray experiments rely on the core principle that transcript abundance can be deduced by measuring the amount of hybridization of labeled RNA to a complementary probe. The idea of a microarray is simply to lay down a field of thousands of these probes in perhaps a 5 sq cm area, where each probe represents the complement of at least a part of a transcript that might be expressed in a tissue. Once the microarray is constructed, the target mRNA population is labeled, typically with a fluorescent dye, so that hybridization to the probe spot can be detected when scanned with a laser. The intensity of the signal produced by 1,000 molecules of a particular labeled transcript should be twice as bright as the signal produced by 500 molecules and, similarly, that produced by 10,000 molecules half as bright as one produced by 20,000 molecules. So a microarray is a massively parallel way to survey the expression of thousands of genes from different populations of cells. Trivially, if fluorescence is observed for a gene in one population but not another, the gene can be inferred to be on or off, respectively. With appropriate replication, normalization, and statistics, though, quantitative differences in abundance as small as 1.2-fold can readily be detected. The output of all microarray hybridizations is ultimately a series of numbers, which covers a range of almost four orders of magnitude, from perhaps one transcript per ten cells to a few thousand transcripts per cell (Velculescu 1999).

It is the comparison of gene expression profiles that is usually of most interest. This is because the visualization is done at the level of transcript abundance, but just seeing a transcript does not guarantee that the protein is produced or functional. If, however, a difference in transcript abundance is observed between two or more conditions, it is natural to infer that the difference might point to an interesting biological phenomenon.

**Figure 1:**
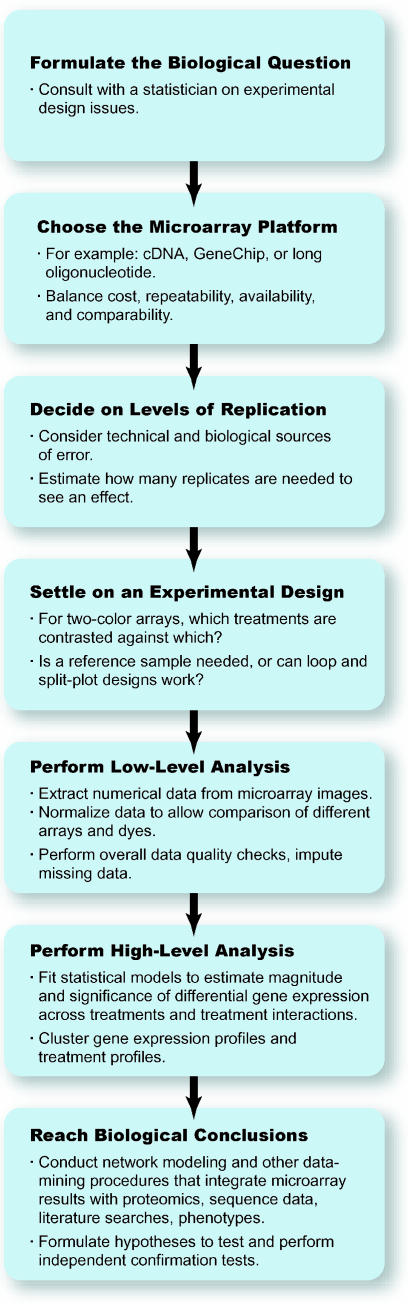
Flow Diagram of Gene Expression Profiling

A general approach to performing gene expression profiling experiments is indicated as a flow diagram in Figure 1. Having performed the experiment, quality control checks, statistical analysis, and data-mining are performed. More and more, investigators are interested not just in asking how large the magnitude of an expression difference is, but whether it is significant, given the other sources of variation in the experiment. Similarly, we might want to evaluate whether some subset of genes show similar expression profiles and so form natural clusters of functionally related genes. Or we may combine expression studies with genotyping and surveys of regulatory sequences to investigate the mechanisms that are responsible for similar profiles of gene expression. Finally, all of the expression inferences must be integrated with everything else that is known about the genes, culled from text databases and proteomic experiments and from the investigator's own stores of biological insight.

## Fishing for Hypotheses

The ability to survey transcript abundance across an ever-increasing range of conditions gives geneticists a fresh look at their cellular systems, in many cases providing a more holistic view of the biology, but at the same time feeding back into the classical hypothetico-deductive scientific framework. The technology has rapidly advanced beyond the simple application of fishing for candidate genes and now sees applications as diverse as clinical prediction, ecosystem monitoring, quantitative mapping, and dissection of evolutionary mechanisms.

Two of the better-known examples of the interplay between microarray profiling and hypothesis testing are provided by the studies of Ideker et al. (2001) and Toma et al. (2002). The latter authors profiled the difference in expression between strains of flies that had been divergently selected for positive and negative geotaxis, a supposedly complex behavior relating to whether flies prefer to climb or stay close to the ground. They identified two dozen differentially expressed genes, several of which were represented by mutant or transgenic stocks that allowed tests of the effect of gene dosage on behavior. At least four of the candidate genes indeed quantitatively affect geotaxis. Ideker et al. (2001) took this approach a step further in arguing for a four-step iterative feedback between profiling, identifying candidate genes, knocking them out, and then profiling once more. They showed how thoughtful experimentation can considerably enhance our understanding of genetic regulatory pathways such as the yeast galactose response.

Much excitement has been generated recently by the potential for clinical applications of gene expression profiling in relation to complex diseases such as cancer, diabetes, aging, and response to toxins. An early foray into this realm was provided by Alizadeh et al. (2000), who demonstrated that diffuse large B-cell lymphomas have two major subtypes defined by molecular profiles. Whereas it is difficult to predict clinical outcome on the basis of histology, these profiles define a set of genes that provide quite a strong indicator of long-term survival. Similarly, van't Veer et al. (2002) have described a “poor prognosis” signature in breast cancer biopsies from young women prior to the appearance of metastases in the lymph nodes. Much statistical and empirical work remains to be done before these tools see clinical application, but the idea that gene expression integrates signals from the genotype and environment provides potent motivation for studying disease with microarrays.

A good example of the ability of microarray analyses to simply surprise us is provided by the study reported in this issue of *PLoS Biology* by DeRisi and colleagues (Bozdech et al. 2003). They reasoned that profiling transcript abundance throughout the erythrocyte phase of the lifecycle of the malaria parasite Plasmodium falciparum might identify a handful of genes that are induced at critical times and hence might be novel drug targets. Employing very careful staging, a platform with low experimental noise, and appropriate statistical procedures, they discovered an extremely tight molecular lifecycle within the organism. Families of functionally related genes are induced as a unit, one after another, in a tightly orchestrated rhythm that testifies to incredible integration of the physiology of the parasite. They show that with microarray analysis it is possible to model the physiology and biochemistry of the pathways instead of just targeting a few genes.

In the coming years, expect to see microarrays developed for an extremely diverse range of organisms and applied to an even wider range of questions, from parasitology to nutritional genomics. Consensus on a core set of statistical options will likely emerge, as will agreement on data quality standards. Applications will encompass defining gene function; inferring functional networks and pathways; understanding how variation is distributed among individuals, populations, and species; and developing clinical protocols relating to cancer prognosis and detection of toxin exposure. Similar profiling methods for proteins and metabolites will attract just as much attention as functional genomics, building on the foundations laid by genome sequencing.

## References

[pbio-0000015-Alizadeh1] Alizadeh AA, Eisen MB, Davis RE, Ma C, Lossos IS (2000). Distinct types of diffuse large B-cell lymphoma identified by gene expression profiling. Nature.

[pbio-0000015-Bozdech1] Bozdech Z, Llinás M, Pullium BL, Wong ED, Zhu J (2003). The transcriptome of the intraerythrocytic developmental cycle of Plasmodium falciparum. PLoS Biol.

[pbio-0000015-Ideker1] Ideker T, Thorsson V, Ranish J, Christmas R, Buhler J (2001). Integrated genomic and proteomic analyses of a systematically perturbed metabolic network. Science.

[pbio-0000015-Toma1] Toma DP, White KP, Hirsch J, Greenspan RJ (2002). Identification of genes involved in Drosophila melanogaster geotaxis, a complex behavioral trait. Nat Genet.

[pbio-0000015-vantVeer1] van't Veer LJ, Dai H, van de Vijver MJ, He YD, Hart AA (2002). Gene expression profiling predicts clinical outcome of breast cancer. Nature.

[pbio-0000015-Velculescu1] Velculescu VE (1999). Tantalizing transcriptomes: SAGE and its use in global gene expression analysis. Science.

